# Anti-Inflammatory and Antimicrobial Activity of Silver Nanoparticles Green-Synthesized Using Extracts of Different Plants

**DOI:** 10.3390/nano14171383

**Published:** 2024-08-25

**Authors:** Amr Mohamed, Marwa Dayo, Sana Alahmadi, Samah Ali

**Affiliations:** 1 Chemistry Department, College of Science, Taibah University, Al-Madinah Al-Munawarah 42353, Saudi Arabia; marwadayo1998@gmail.com (M.D.); sahamade@taibahu.edu.sa (S.A.); 2 The Higher Institute of Optics Technology (HIOT), Heliopolis, Cairo 17361, Egypt; 3 The National Organization for Drug Control and Research, Giza 12622, Egypt

**Keywords:** silver nanoparticles, *Ginkgo biloba*, *Cichorium intybus*, *Adiantum capillus-veneris*, *Rosmarinus officinalis*, anti-inflammatory and antimicrobial

## Abstract

In this study, an easy, efficient, economical, and eco-friendly green bio-synthesis method was utilized to synthesize silver nanoparticles (AgNPs) using the extracts of four plants: *Ginkgo biloba*, *Cichorium Intybus*, *Adiantum Capillus-Veneris*, and *Rosmarinus Officinalis*. The synthesis of AgNPs was confirmed by using a uv-vis spectrometer, which showed distinct surface plasmon resonance (SPR) bands. The surface of AgNPs was characterized using scanning electron microscopy and Fourier-transform infrared spectroscopy. The anti-inflammatory activity of Tenoxicam/Meloxicam-loaded AgNPs has been studied using the inhibition of albumin denaturation method. Tenoxicam-loaded AgNPs showed higher % Inhibition, but Meloxicam-loaded AgNPs showed lower % Inhibition. Furthermore, the AgNPs showed excellent antimicrobial activity on both Gram-negative and Gram-positive bacteria.

## 1. Introduction

Due to their unique and important features relative to bulk materials, nanoparticles (NPs) have received a lot of attention over the years and have frequently been the focus of research. NPs can be defined as extremely small materials, particularly on the nanoscale 1–100 nm, possessing at least one external dimension in that range [1]. They are being used in several fields, most importantly being the industrial, mechanical, agricultural, environmental, and medical fields [2].

Silver nanoparticles (AgNPs) are a type of NPs that are of great interest in research due to their distinct optical, chemical, and physical properties [3], in addition to their special and unique potential biological activities like their antibacterial [4], antifungal [5], antioxidant, angiogenic [6], anticancer [7], antifouling [8], antiparasitic [9], and anti-inflammatory uses [10], etc. Therefore, they have a wide scope of applications in pharmacology, chemistry, agriculture, food, and textile industries [11]. Many approaches have been used to synthesize AgNPs, which can be classified into physical, chemical, and biological approaches [12]. Physical methods require complex and expensive equipment, high energy, large spaces, and they are mostly time-consuming [13]. The chemical methods require costly precursors and the use of toxic or hazardous solvents and chemical reductants [14], and the residual solutions from the synthesis are usually acidic, alkaline, poisonous, and harmful to the environment [15]. On the other hand, the biological approach is an easy, safe, economical, and eco-friendly method that utilizes biological sources like microorganisms, bacteria, algae, fungi, and plants [16]. These sources act as the reducing agents and caping ligands/stabilizing agents to produce AgNPs [17], and the synthesis is completed without using and generating harsh, hazardous, or toxic chemicals [18], which offers a new and potential alternative to the conventional methods [19].

Plant-based synthesis of AgNPs has attracted much more attention recently, and has been more favourable than the other biological methods because of its easy and effortless synthetic procedure and its suitability for large-scale production [20]. Several parts of plants, including stems, roots, latex, leaves, seeds, flowers, peels, and fruits, have been used to synthesize AgNPs [21]. Plant extracts usually contain various biomolecules such as alkaloids, terpenoids, phenols, flavonoids, tannins, and quinines, etc., that act as reducing and stabilizing agents for Ag^+^, and they easily adsorb on the surface of AgNPs, enhancing their stability [22]. Optimizing the shape, size, and distribution of the produced AgNPs could be carried out by controlling the synthesis method, reducing and stabilizing factors, extract and precursor concentrations, and parameters such as pH and temperature [23]. The general procedure for the synthesis of AgNPs using plant extracts is performed simply by mixing the plant extract with the silver metal salt solution at room temperature. The synthesis is usually completed in a short period of time [24].

**Figure 1:**
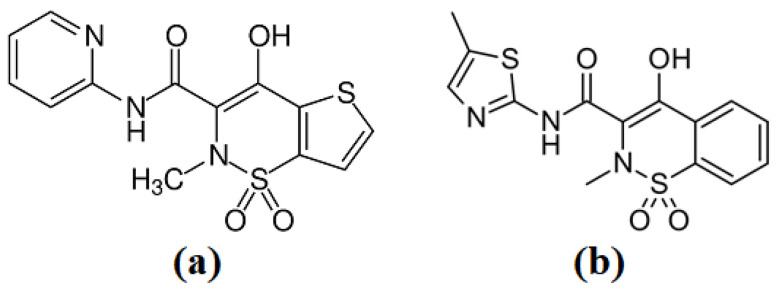
The chemical structure of (**a**) Tenoxicam drug and (**b**) Meloxicam drug [26,27].

Tenoxicam (TNX) and Meloxicam (MLX) with the structures shown in Figure 1 are within the same drug class. They are effective nonsteroidal anti-inflammatory drugs and active ingredients of the oxicam chemical class for treating ankylosing spondylitis, osteoarthritis, rheumatoid arthritis, and various rheumatic conditions. Their efficacy is like other non-steroidal anti-inflammatory drugs, and are well tolerated as piroxicam and probably better tolerated than indomethacin, ketoprofen, and diclofenac. Their daily dose is usually 10–20 mg and 7.5–15 mg, respectively [25].

The aim of this work is to synthesize AgNPs using the plant extracts of *Ginkgo biloba (GB)*, *Cichorium intybus (CI)*, *Adiantum capillus-veneris (AC)*, and *Rosmarinus officinalis (RO)* using a single easy, economical, and eco-friendly method. Moreover, the anti-inflammatory and antimicrobial activity of the synthesized AgNPs using each plant’s extract has been studied.

## 2. Materials and Methods

### 2.1. Instrumentation

The pH of the solutions was measured by using a pH meter (HI 221, Hanna-Instruments, Eibar, Spain) with an Ag/AgCl electrode. A digital orbital shaker (Stuart SSL-1, Carl Roth, Karlsruhe, Germany) was used for sorption experiments, and a centrifuge (2010, Kubota, Tokyo, Japan) was used to obtain the AgNPs. The absorbance measurements were carried out using a uv-vis spectrophotometer (Evolution 201, Thermo Scientific, MA, USA) with matching glass cells (10 mm). The morphology of AgNPs was studied using a scanning electron microscope (SEM) model (1400, Jeol, Tokyo, Japan). The surface functional groups of AgNPs were studied using an IR spectrometer (FTIR-8400S, Shimadzu, Kyoto, Japan).

### 2.2. Materials and Reagents

All reagents were of analytical grade. Silver nitrate (AgNO_3_) was obtained from Sigma-Aldrich Chemicals. Plant materials were obtained from a commercial store in Saudi Arabia. The Tenoxicam (TNX) drug was supplied by Ramdev Chemical PVT. LTD., Maharashtra, India. The Meloxicam (MLX) drug with the brand name Coxicam was purchased from a local pharmacy. Double-distilled water was used in all preparations.

### 2.3. Methods

#### 2.3.1. Preparation of Plant Powders

**Figure 2:**
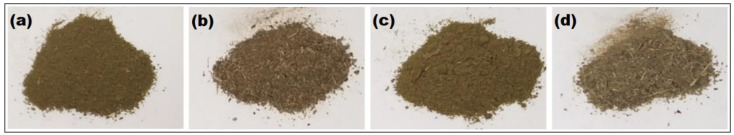
Plant powders: (**a**) *Ginkgo biloba*, (**b**) *Cichorium intybus*, (**c**) *Adiantum capillus-veneris*, and (**d**) *Rosmarinus officinalis*.

The plants (GB, AC, CI, and RO) obtained from a commercial store were washed with water to remove dust and other contaminants, followed by washing with double-distilled water. After washing, they were left to dry in shadow at room temperature. Upon complete drying, they were ground using a blender to obtain powders, as shown in Figure 2, which were stored in a container in a dry and dark place at room temperature until use.

#### 2.3.2. Preparation of Plant Extracts

**Figure 3:**
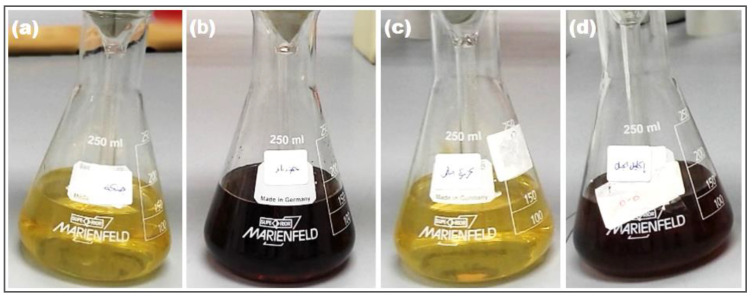
Plant extracts: (**a**) *Ginkgo biloba*, (**b**) *Cichorium intybus*, (**c**) *Adiantum capillus-veneris*, and (**d**) *Rosmarinus officinalis*.

The plants’ extracts of (GB, AC, CI, and RO) were prepared by suspending 2.0 g of each plant’s powder in 100 mL of double-distilled water; then, using a digital orbital shaker, the suspended solution was shaken at 200 rpm for 2 h. After that, the solution was filtered using S&S filter paper grade 602 H. The obtained filtrates, shown in Figure 3, were stored in a refrigerator at 4–6 °C until use.

The 2% aqueous solutions of the extracts of (GB, AC, CI, and RO) plants were prepared by adding 2 mL of each of the previously prepared extracts to 100 mL volumetric flasks and diluting them with double-distilled water.

### 2.4. Synthesis of Silver Nanoparticles

AgNPs were synthesized by mixing 0.01 M silver nitrate solution with multiple volumes of plant extracts and shaking for different time periods between 15 and 120 min at 200 rpm. The synthesized colloidal AgNPs were characterized using a uv-vis spectrophotometer. The synthesized AgNPs using the four plants’ extracts were isolated from the colloidal solution by centrifuging them at 4000 rpm for 15 min; then, the supernatant solution was discarded, and the solid was washed with double-distilled water three times. The solid with a small amount of distilled water was transferred to a pre-weighed empty and dry beaker and left to dry at room temperature to obtain the solid AgNPs.

### 2.5. Loading of AgNPs with an Anti-Inflammatory Drug

AgNPs were loaded with an anti-inflammatory drug by mixing 0.5 mL of 0.1 mg/mL drug solution (Tenoxicam/Meloxicam) with 0.5 mL of 0.59 mg/mL of the synthesized AgNPs; then, the solutions were shaken at 200 rpm for 1 h. Then, they were investigated using a uv-vis spectrophotometer.

### 2.6. Anti-Inflammatory Activity of Tenoxicam/Meloxicam-Loaded AgNPs

The anti-inflammatory activity of Tenoxicam/Meloxicam-loaded AgNPs synthesized using the four plants’ extracts was investigated using the method of inhibition of protein denaturation under heat stress following the procedure reported by Sirry et al. [28], with minor modifications. The method was performed by adding 0.2 mL of fresh albumin (egg white) to 2.8 mL of phosphate buffer of pH 7.4 followed by adding 0.5 mL of Tenoxicam/Meloxicam-loaded AgNPs. For quality control purposes, 0.2 mL of fresh albumin (egg white) was added to 2.8 mL of phosphate buffer of pH 7.4 followed by adding 0.5 mL of distilled water. The solutions were incubated in a water bath at 37 ± 2 °C for 15 min, and then the temperature was raised to 70 °C for 5 min. After the solutions were cooled to room temperature, their absorbance was measured using a uv-vis spectrophotometer at 660 nm. The percentage of inhibition of protein denaturation (%*I*) under heat stress was calculated using Equation (1):

(1) $$\%I=\frac{Ac-As}{Ac}\times 100$$

where *Ac* and *As* is the absorbance of control and sample, respectively.

### 2.7. Antimicrobial Activity of AgNPs

The antimicrobial activity of AgNPs was tested against microbial strains provided by the Regional Center for Mycology and Biotechnology (RCMB), Al-Azhar University, Cairo, Egypt. Gram-positive (*Staphylococcus aureus*) and Gram-negative (*Escherichia coli*) bacteria were used with a modified well diffusion method [29]. The AgNPs with the concentration of 10 mg/mL were prepared using DMSO as a solvent. In total, 100 μL of bacteria was grown in 10 mL of fresh media until reaching around 10^8^ cells/mL of bacteria. The suspension was spread onto agar plates corresponding to the broth in which they were preserved and tested for their susceptibility, and then wells of 6 mm diameter were made in which 100 µL of each AgNPs were added. The plates were incubated at 37 °C for 24 h. After incubation, the growth of the microorganisms was observed, and the diameters of the resulting inhibition zones were measured in millimetres and used as the standard for the antimicrobial activity. For the negative controls, DMSO was used, while the positive controls were executed by using gentamycin as the standard antibacterial drug.

## 3. Results and Discussions

### 3.1. Characterization of Silver Nanoparticles

**Figure 4:**
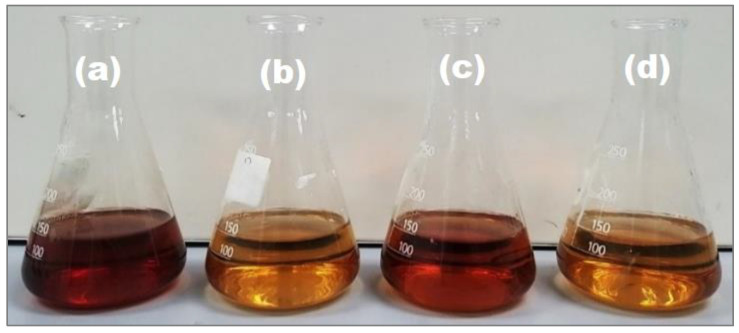
Colour change of reaction mixtures: (**a**) GB-AgNPs, (**b**) CI-AgNPs, (**c**) AC-AgNPs, and (**d**) RO-AgNPs.

The general reaction that leads to the formation of AgNPs is the reduction of silver salt ions, Ag^+^, to metallic Ag^0^, which is typically a fast process [30]. The different components of the plant extracts, such as flavonoids, ketones, aldehydes, carboxylic acids, tannins, proteins, and phenolic compounds, act as the reducing and stabilizing agents [31]. The functional groups in these compounds, such as phenolic hydroxyl, carboxyl, and carbonyl groups, could be the reason for the reduction and stabilization process [32]. The synthesis of AgNPs was indicated first by a colour change in the reaction mixture (plant extract + AgNO_3_). The colour change is due to the formation of AgNPs [33]; they are known to exhibit a yellow or brown colour due to the excitation of their surface plasmon resonance (SPR) vibrations [34]. AgNPs were successfully synthesized using the extracts of *Ginkgo biloba*, *Cichorium intybus*, *Adiantum capillus-veneris*, and *Rosmarinus officinalis* plants, and the colour change of the reaction mixtures is shown in Figure 4. The solutions exhibit distinct colours due to the morphology of the resultant NPs, quantum size confinement, and/or surface plasmon [10].

#### 3.1.1. UV–Visible Spectroscopic Analysis of AgNPs

**Figure 5:**
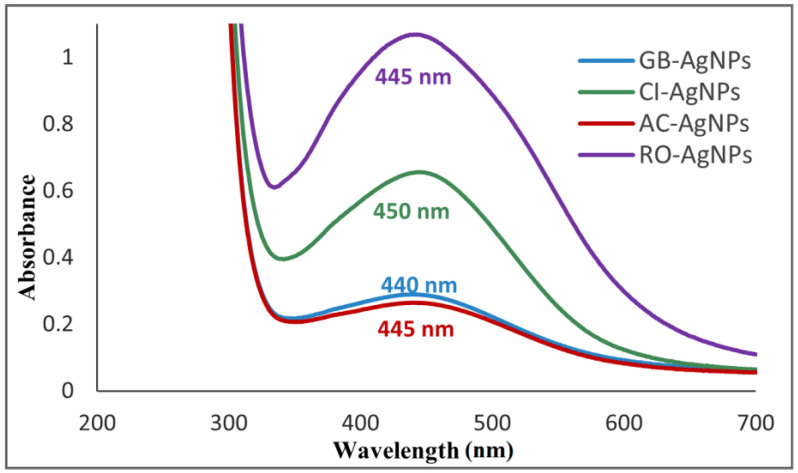
Uv-vis spectra of bio-synthesized AgNPs.

The bio-synthesized AgNPs (GB-AgNPs, CI-AgNPs, AC-AgNPs, and RO-AgNPs) (5 mL of 0.01 M AgNO_3_ with 7 mL of each extract (5:7 ratio)) were characterized using a uv-vis spectrophotometer, which showed SPR bands at 445, 440, 450, and 445 nm, respectively, as shown in Figure 5. The wavelength range 400–470 nm corresponds to AgNPs production. The peak locations in the spectra are primarily affected by the shape of NPs, temperature, and the dielectric constant of the medium, suggesting poly dispersibility of the synthesized AgNPs [34], and the different shapes of the peaks could be due to the different reducing agents of the plants involved in the synthesis of the AgNPs [35].

#### 3.1.2. Morphological Study Using SEM

**Figure 6:**
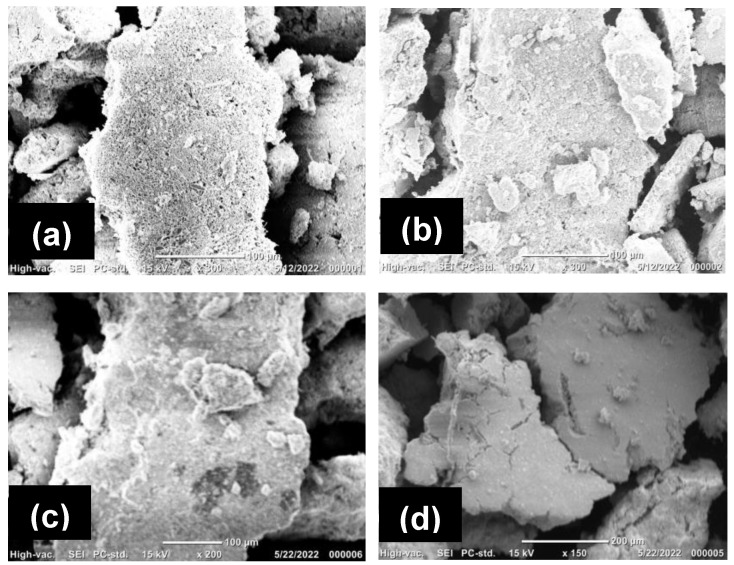
SEM images of AgNPs: (**a**) GB-AgNPs, (**b**) CI-AgNPs, (**c**) AC-AgNPs, and (**d**) RO-AgNPs.

The morphology of AgNPs plays a crucial part in their properties and potential applications in nanotechnology and biomedicine. In this study, using scanning electron microscopy (SEM), the morphology of the green-synthesized AgNPs was examined. The SEM images in Figure 6 show highly aggregated clusters of AgNPs; they were observed to be spherical in shape, with an average size of 80–100 nm. The results are in agreement with previous studies [36,37].

#### 3.1.3. Fourier-Transform Infrared Spectroscopy (FT-IR)

**Figure 7:**
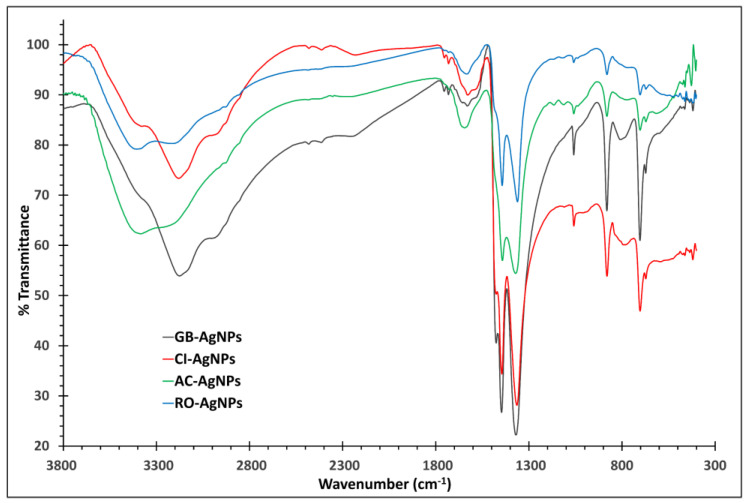
FT-IR images of GB-AgNPs, CI-AgNPs, AC-AgNPs, and RO-AgNPs.

The surface of the AgNPs was characterized using FT-IR spectroscopy in the range of 400–4000 cm^−1^, which was carried out to distinguish the possible compounds and biomolecules involved in the synthesis of AgNPs; the results are presented in Figure 7, which shows the similarity of the detected bands and functional groups in the spectra. Table 1 summarizes the exact functional groups’ peaks for each spectrum. The broad band around 3000–3500 cm^−1^ corresponds to the overlapped hydroxyl O-H and amine N-H groups’ stretching vibrations. The band around 2900 cm^−1^ represents the C-H stretching band, and the two peaks found around 1630–1400 cm^−1^ correspond to the carbonyl C=O and alkene C=C group, respectively. The stretching bands of C-N and C-O can be observed around 1360–1440 cm^−1^. The small peaks in the range from 880 to 460 cm^−1^ represent the aromatic C-H bending vibrations. Most of the vibration bands detected were similar to previous reports [38]. The assigned vibration bands represent many compounds, including proteins, phenols, terpenoids, flavonoids, carboxylic acids, alcohols, aldehydes, ketones, amino acids, esters, and ethers. The presence of these compounds confirms their binding abilities to the silver metal to form metal nanoparticles, along with the prevention of agglomeration and the stabilization of the reaction medium [39].

### 3.2. Effect of Reaction Time

**Figure 8:**
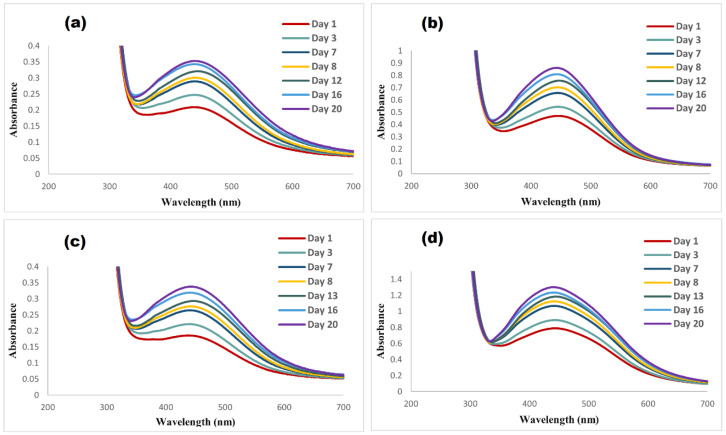
Effect of reaction time on the absorbance bands of (**a**) GB-AgNPs, (**b**) CI-AgNPs, (**c**) AC-AgNPs, and (**d**) RO-AgNPs.

Reaction time is an important factor for synthesizing nanoparticles [40]. The results shown in Figure 8 indicate an increase in the absorbance with an increase in the reaction time, which correlates with an increase in AgNPs concentration in the solution [41]. In this study, 5 mL of 0.01 M AgNO_3_ were mixed with 7 mL of each extract (5:7 ratio). The maximum absorption was found on day 7. The wavelengths of the bands remained unchanged until day 8, where the wavelengths red-shifted, and the red-shift increased by increasing the reaction time. The shifting of the wavelengths of the AgNPs may be due to the increase in size and the aggregations of colloidal nanoparticles, along with the presence of high concentrations of stabilizing agents in the plant extracts [42].

### 3.3. Effect of Extract and Silver Ion Volumes

**Figure 9:**
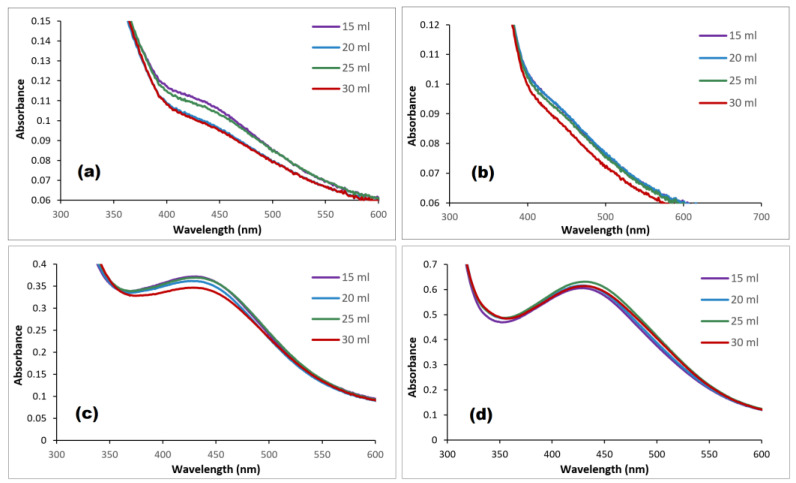
Effect of plant extract’s volume on the formation of (**a**) GB-AgNPs, (**b**) CI-AgNPs, (**c**) AC-AgNPs, and (**d**) RO-AgNPs.

**Figure 10:**
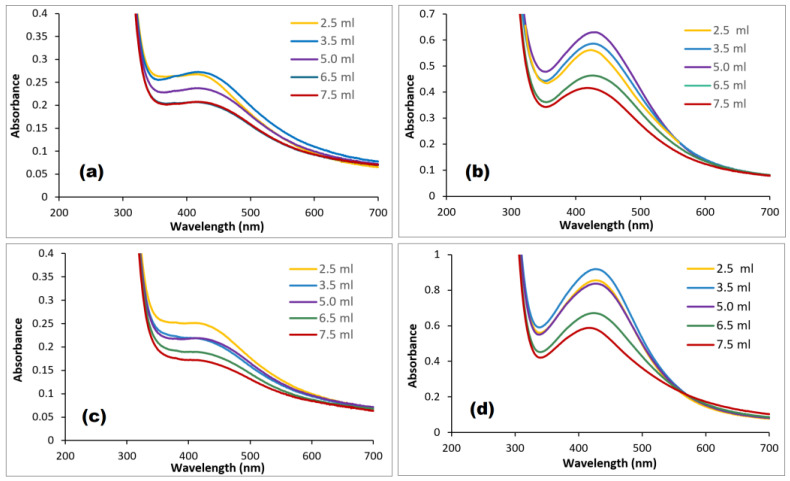
Effect of silver ion’s concentration on the absorbance bands of (**a**) GB-AgNPs, (**b**) CI-AgNPs, (**c**) AC-AgNPs, and (**d**) RO-AgNPs.

The plant extracts contain high concentrations of compounds that act as reducing and capping agents, which may influence the formation of AgNPs. Therefore, variable volumes of (GB, AC, CI, and RO) plant extract were added to a 0.01 M AgNO_3_ solution; the surface plasmon resonance (SPR) absorption bands are shown in Figure 9. The formation of AgNPs was enhanced by increasing the plant extract’s volume [43]. The maximum yield was obtained by mixing 15, 15, 20, and 25 mL of GB, AC, CI, and RO 2% extract, respectively, with 0.01 M AgNO_3_. The wavelength of SPR bands did not change by changing the extracts’ volumes, which indicates that the diameter of AgNPs remained the same within these ranges of extract concentrations. However, increasing the volume of silver ion solution did change the SPR bands: starting from 6.5 mL of 0.01 M AgNO_3_, the bands were red-shifted [44], as shown in Figure 10.

### 3.4. In Vitro Anti-Inflammatory Activity of Tenoxicam/Meloxicam-Loaded AgNPs

The anti-inflammatory activity of the green-synthesized AgNPs has been studied through the protein denaturation method. Protein denaturation is a process in which proteins (nucleic acids) lose their quaternary, tertiary, and secondary structures due to some external stress or compounds such as an organic solvent, concentrated inorganic salt, strong acid or base, agitation, and radiation or heat, which is the reason for inflammation [45]. Tenoxicam and Meloxicam (TNX/MLX) drugs reduce the denaturation under these stresses and cause an anti-inflammatory effect [46].

**Figure 11:**
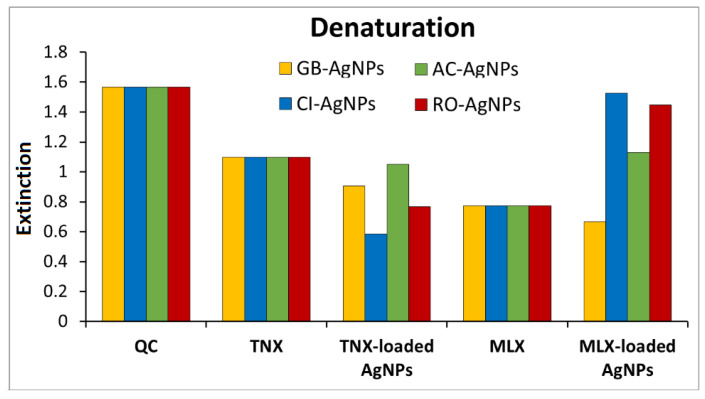
Denaturation of albumin protein in different samples.

TNX/MLX-loaded AgNPs anti-inflammatory activity was studied against TNX/MLX anti-inflammatory drug alone. The albumin denaturation is shown in Figure 11, which was extremely high in natural conditions, but it decreased slightly with the TNX drug, and decreased to half with the MLX drug. The denaturation was decreased even more in the presence of TNX-loaded AgNPs, but it decreased slightly in the presence of MLX-loaded AgNPs, which could be due to the interferences in the pharmaceutical drug. The albumin denaturation % inhibition results are represented in Table 2. The Tenoxicam drug showed inhibition by 29.95%, whereas TNX-loaded AgNPs showed 42.1%, 62.58%, 32.95%, and 50.96% inhibition for GB-AgNPs, CI-AgNPs, AC-AgNPs, and RO-AgNPs, respectively, which is significantly high in the case of CI-AgNPs and RO-AgNPs, and considerably higher for GB-AgNPs and AC-AgNPs when compared to the TNX drug. The Meloxicam drug showed inhibition by 50.51%, whereas MLX-loaded AgNPs showed 57.41%, 2.55%, 27.84%, and 7.66% inhibition for GB-AgNPs, CI-AgNPs, AC-AgNPs, and RO-AgNPs, respectively, which is slightly higher for GB-AgNPs and is lower for CI-AgNPs, AC-AgNPs, and RO-AgNPs when compared to the MLX drug.

### 3.5. Antimicrobial Activity

The antimicrobial activity of the green-synthesized AgNPs was investigated on two pathogenic microorganisms (*E. coli* and *S. aureus*) using the agar diffusion technique. The well diffusion method shows the amount of susceptibility in the pathogenic microorganisms; therefore, if an organism is sensitive to the chemical, it will not grow in the vicinity of the well it was placed in. The no-growth area is called the zone of inhibition or the clear zone. The size of the clear zone is proportional to the inhibition of the tested compound. The green-synthesized AgNPs showed great antimicrobial activity against both Gram-negative and Gram-positive bacterial strains. The mean zones of inhibition in mm produced on the pathogenic microorganisms containing AgNPs suspension are presented in Table 3. The CI-AgNPs and AC-AgNPs showed slightly higher inhibition against *S. aureus* Gram-negative bacteria than GB-AgNPs and RO-AgNPs, whereas CI-AgNPs and RO-AgNPs showed slightly higher inhibition against *E. coli* Gram-positive bacteria than GB-AgNPs and AC-AgNPs. This can be attributed to the difference in cell membrane permeability between the two bacterial strains, which is significantly affected by a variety of complex factors, including the zeta potential on the membrane, the lipophobicity of the cell membrane, the thickness of the membrane and its surrounding layers, and the chemical composition of the antimicrobial drug [47,48,49,50,51]. These results confirm the high antimicrobial potential of the green-synthesized silver nanoparticles.

## 4. Conclusions

AgNPs were successfully green-synthesized by using the extracts of four plants (*Ginkgo biloba*, *Cichorium intybus*, *Adiantum capillus-veneris*, and *Rosmarinus officinalis*) following a single elementary, efficient, economical, and eco-friendly synthesis method. They were investigated using uv-vis spectroscopy, and showed SPR bands in the range of 440–450 nm. The surface of AgNPs was studied using SEM and FT-IR, which confirmed the synthesis. Then, the anti-inflammatory activity of Tenoxicam-loaded AgNPs was studied usinh the inhibition of albumin denaturation method. The loaded AgNPs (GB-AgNPs, CI-AgNPs, AC-AgNPs, and RO-AgNPs) showed the % inhibition of 42.1%, 62.58%, 32.95%, 50.96% respectively, which was significantly higher than the 29.95% of the standard Tenoxicam drug. The process was also applied to the pharmaceutical drug Meloxicam, and the results were considerably great for GB-AgNPs but not as good for CI-AgNPs, AC-AgNPs, and RO-AgNPs, as they showed less % inhibition than the standard Meloxicam drug. Additionally, the antimicrobial activity was investigated against *E. coli* Gram-positive and *S. aureus* Gram-negative bacteria, and the green-synthesized AgNPs showed a high antimicrobial capability.

## Figures and Tables

**Table 1:** FT-IR spectral peaks and the corresponding functional groups.

Functional Group	GB-AgNPs	CI-AgNPs	AC-AgNPs	RO-AgNPs
(N-H) Stretching	3350 cm^−1^	3390 cm^−1^	3384 cm^−1^	3406 cm^−1^
(O-H) Stretching	3175 cm^−1^	3181 cm^−1^	3200 cm^−1^	3214 cm^−1^
(C-H) Stretching	2920 cm^−1^	2920 cm^−1^	2850 cm^−1^	2950 cm^−1^
(C=O) Stretching	1631 cm^−1^	1627 cm^−1^	1644 cm^−1^	1632 cm^−1^
(C=C) Stretching	1540 cm^−1^	1475 cm^−1^	1644 cm^−1^	1632 cm^−1^
(C-O) Stretching	1446 cm^−1^	1445 cm^−1^	1442 cm^−1^	1443 cm^−1^
(C-N) Stretching	1369 cm^−1^	1364 cm^−1^	1371 cm^−1^	1361 cm^−1^
Aromatic C-H Bending	881–672 cm^−1^	880–672 cm^−1^	880–671 cm^−1^	880–671 cm^−1^

**Table 2:** % Inhibition of samples.

Material	Tenoxicam	TNX-Loaded AgNPs			
		GB-AgNPs	CI-AgNPs	AC-AgNPs	RO-AgNPs
**% Inhibition**	29.9489	42.1456	62.5798	32.9502	50.9578
**Material**	**Meloxicam**	**MLX-Loaded AgNPs**			
		**GB-AgNPs**	**CI-AgNPs**	**AC-AgNPs**	**RO-AgNPs**
**% Inhibition**	50.5108	57.4074	2.5543	27.8416	7.6628

**Table 3:** Inhibition zone diameter (mm) of the AgNPs against Gram-positive and Gram-negative bacteria.

Test Microorganism	Control Gentamycin	AgNPs Samples			
		GB-AgNPs	CI-AgNPs	AC-AgNPs	RO-AgNPs
Gram-Positive bacteria: *Staphylococcus aureus* ATCC 25923	27	14	16	16	15
Gram-Negative bacteria: *Escherichia coli* ATCC 25922	31	18	19	18	19

## Data Availability

The data presented in this study are available on request from the corresponding author.
