# Correction: Mohamed et al. Anti-Inflammatory and Antimicrobial Activity of Silver Nanoparticles Green-Synthesized Using Extracts of Different Plants. *Nanomaterials* 2024, *14*, 1383

**DOI:** 10.3390/nano16010070

**Published:** 2026-01-04

**Authors:** Amr Mohamed, Marwa Dayo, Sana Alahmadi, Samah Ali

**Affiliations:** 1 Chemistry Department, College of Science, Taibah University, Al-Madinah Al-Munawarah 42353, Saudi Arabia; marwadayo1998@gmail.com (M.D.); sahamade@taibahu.edu.sa (S.A.); 2 The Higher Institute of Optics Technology (HIOT), Heliopolis, Cairo 17361, Egypt; 3 The National Organization for Drug Control and Research, Giza 12622, Egypt

In the original publication [1], reference [43] has been retracted prior to the publication. Concerns were raised, and the authors want to replace it with the following reference:

43. Phukan, K.; Devi, R.; Chowdhury, D. Green Synthesis of Gold Nano-bioconjugates from Onion Peel Extract and Evaluation of their Antioxidant, Anti-inflammatory, and Cytotoxic Studies. *ACS Omega* **2021**, *8*, 17811–17823.

The original publication also includes the newly added GA.

The authors state that the scientific conclusions are unaffected. This correction was approved by the Academic Editor. The original publication has also been updated.
